# Immunogenicity as a Predictor of Influenza Vaccine Efficacy: A Systematic Review

**DOI:** 10.3390/vaccines13080859

**Published:** 2025-08-14

**Authors:** André Miguel Martins, Luis Félix Valero Juan, Marlene Santos, João P. Martins

**Affiliations:** 1 LAQV/REQUIMTE, Escola Superior de Saúde, Instituto Politécnico do Porto, Rua Dr. António Bernardino de Almeida, 4200-072 Porto, Portugal; 2 Medicina Preventiva y Salud Pública, Facultad de Medicina, Universidad de Salamanca, 37007 Salamanca, Spain; luva@usal.es; 3 Molecular Oncology & Viral Pathology, IPO-Porto Research Center, Portuguese Institute of Oncology, Rua Dr. António Bernardino de Almeida, 4200-072 Porto, Portugal; 4 Escola Superior de Saúde, Instituto Politécnico do Porto, Rua Dr. António Bernardino de Almeida, 4200-072 Porto, Portugal; jom@ess.ipp.pt; 5 CEAUL—Centro de Estatística e Aplicações, Faculdade de Ciências, Universidade de Lisboa, 1749-016 Lisboa, Portugal

**Keywords:** influenza, influenza vaccine, efficacy, immunogenicity

## Abstract

Background/Objectives: Influenza represents a significant burden on global public health, and vaccination is the most effective strategy to reduce it. The large investment in vaccination programs and the need for adjustments in vaccine serotypes are important reasons for evaluating the influenza vaccine’s efficacy every year. Establishing a relationship between immunogenicity data and efficacy is also crucial for predicting the efficacy of a vaccine during its development. Antibody response measurement is one of the most common methods for evaluating immunogenicity, particularly in vaccines and biologics. The aim of this systematic review was to define a model that relates the immunogenicity of a given vaccine to its efficacy, based on hemagglutination inhibition titer levels. Methods: To achieve this goal, information was gathered from articles linking immunogenicity with the efficacy of the influenza vaccine in the Medline and Scopus databases. Different mathematical models were developed and applied to assess the relationship between HAI titers and the effectiveness of the flu vaccine. This analysis was conducted for the various existing vaccines, for the different influenza virus strains, and for their efficacy in paediatric populations. Results: The r^2^ obtained ranged from 0.2579 to 0.966, which points to the importance of this immunological factor in the efficacy of the influenza vaccine. Conclusions: The efficacy values for titer level 40 confirm the validity of the data provided by Hobson.

## 1. Introduction

Influenza is a respiratory disease resulting from infection with an influenza virus and is highly transmissible in humans [1]. The World Health Organization (WHO) estimates one billion cases of influenza worldwide each year, of which 3–5 million represent severe forms [2]. An estimated number of 650,000 deaths per year result from influenza infection [3]. The most effective way to prevent influenza infection and its complications is through vaccination [4].

Influenza is caused by several RNA viruses from the *Orthomyxoviridae* family. Influenza viruses are classified into four types: A, B, C, and D [5]. The four genera of influenza viruses are identified by antigenic differences in their nucleoproteins and matrix proteins [6]. Only types A, B, and C are associated with infections in humans [7], and type C is very rare [8]. This is why flu vaccines only include influenza A and influenza B. Influenza A is divided into subtypes based on the presence of specific haemagglutinin (HA) and neuroaminidase (NA) proteins on the surface of the virus, and the most common in circulation are A(H1N1) and A(H3N2). Surface glycoproteins (hemagglutinin (HA) and neuroaminidase (NA)) allow the virus to attach to, enter, and exit infected cells [6]. Influenza B comprises two specific lineages: Victoria and Yamagata.

There are two types of vaccines against influenza viruses: inactivated vaccines, including trivalent (TIV) and tetravalent (QIV) designs, and live attenuated virus vaccines (LAIVs) [9].

The response to a vaccine involves both cellular and humoral immunity factors [10,11]. The measurement of HA specific antibodies using hemagglutination inhibition (HAI) and microneutralization (MN) assays remains the primary correlate of protection against influenza [12]. The study by Hobson et al. [13], which evaluated antibodies produced from a natural infection to estimate the HAI titer associated with protection, has classified an HAI titer of at least 1:40 as protective in adults. Different HAI titers have been identified in studies for various degrees of protection. This applies both to children and adults [14,15,16].

Evaluating the effectiveness of the flu vaccine that is used every year is essential both because of the large investment that vaccination programs represent and because of the need for adjustments in vaccine serotypes for them to become more effective in controlling the disease. Many studies have evaluated vaccine efficacy and point out that the concordance between the strains included in the vaccine and the most prevalent strains in circulation is the most influential determining factor for good vaccine efficacy [17,18]. Establishing a relationship between immunogenicity data and efficacy is crucial for predicting the effectiveness of a vaccine during its development.

The main objective of this systematic review was to define a model that relates the immunogenicity of a given vaccine to its efficacy, based on the levels of HAI titers, updating the data by Hobson et al. [13].

## 2. Materials and Methods

### 2.1. Search Strategy and Eligibility Criteria

A comprehensive electronic search of PubMed and Scopus was undertaken to identify studies examining the association between immunogenicity and efficacy. The following search string was used: TITLE-ABS-KEY ((flu OR influenza OR influenzavirus) AND (HAI OR NAI) AND (RCT OR clinical trial OR controlled trial OR efficacy)). Research was carried out up to December 2024, and the study has been registered on the INPLASY platform with the registration number INPLASY202570064.

Studies were included if they met the following criteria: (i) they reported immunogenicity data including HAI titers; (ii) were randomized clinical trials (RCTs); (iii) assessed the association between HAI levels and efficacy against infection, (iv) including studies in which data were available only in graphical form; and (v) were published in English. No restriction on publication date was used, given the limited number of relevant studies.

### 2.2. Outcome Measure

The measurements of stress and protection against influenza infection are associated with the level of HAI titers. Vaccine performance is determined by vaccine efficacy (VE), which is equal to the following:

VE = (1 − RR) × 100

where RR is the relative risk, which is given by the proportion of infections among the vaccinated over the proportion of infections among the unvaccinated. The estimate of the vaccine efficacy was extracted for each observed titer level. When it was not reported, efficacy was calculated by using the proportion of infections among the vaccinated at each titer level as the numerator and the proportion of infections among all the unvaccinated as the denominator in the RR formula.

### 2.3. Data Collection and Analysis

Two authors of this review independently assessed the studies’ eligibility by screening the titles and abstracts. All selected articles from this initial screening were further reviewed for inclusion through full-text assessment. The information from all selected papers was independently extracted into a form that included the following: study design, participants, sample size, description of intervention, outcomes, and quality assessment indicators. WebPlotDigitizer, version 3.4 [19] software was used to digitize plots when analytical data were not available. Discrepancies in study selection were resolved through a consensus.

Models for associating vaccine efficacy with HAI titers were derived through a meta-regression approach. Polynomial (linear, quadratic, and cubic), exponential, and logarithmic models were tested, along with a generalized additive model using cubic regression splines with 4 knots. The weight of each observation was given by the sample size divided by the number of reported VE estimates of each study. When VE estimates were not reported, the weight was calculated by (number of vaccinated for a level of titers/total number of vaccinated) × sample size. The best model was selected using the Bayesian Information Criterion (BIC).

For each strain of influenza virus and type of vaccine, a model was fitted. The different models were applied to the specific population of children, in addition to testing all the information gathered.

The selected models were used to calculate an estimate of the efficacy for HAI titer levels of 1:30, 1:40, and 1:50. The quality of the model fit was evaluated using the coefficient of determination r^2^. Data analysis was conducted in R software [20], including splines [21] and mgcv packages [22].

### 2.4. Quality Assessment

Two authors independently assessed the included studies for risk of bias using validated critical appraisal tools. Inconsistencies were resolved by a third reviewer.

The Cochrane risk-of-bias tool for randomized trials (RoB 2) was used for RCTs [23]. Data were input using the RoB 2 Excel tool (available at https://www.riskofbias.info, accessed on 1 March 2025). The sensitivity analysis was not conducted because no studies with a high risk of bias were identified.

## 3. Results

**Figure 1:**
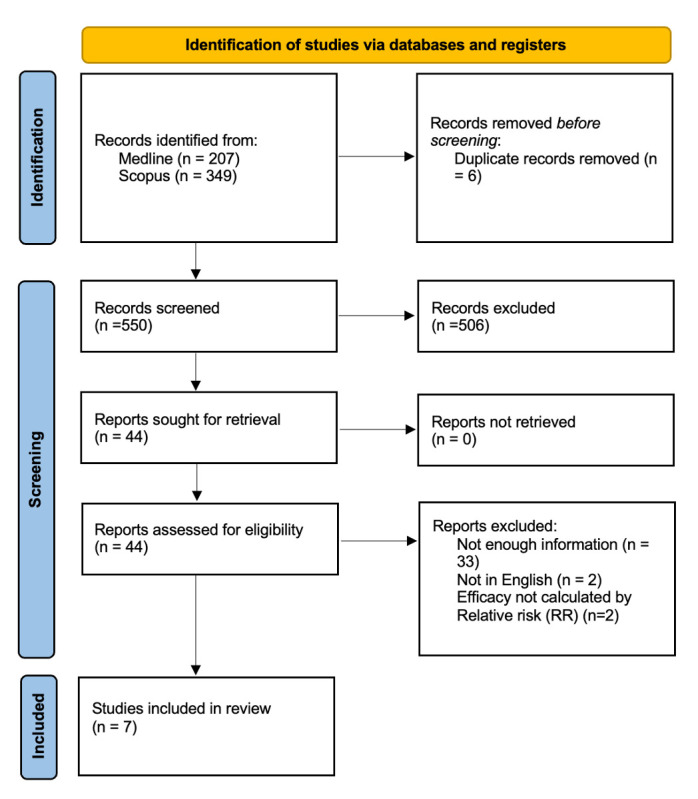
PRISMA study search flow diagram.

The search identified a total of 551 records after removing duplicates. The full-text versions of 44 records were screened for eligibility, 37 of which were excluded. A total of seven papers were included in the study [24,25,26,27,28,29,30]. The selection process is detailed in the PRISMA flowchart (see Figure 1).

The main characteristics of the RCT studies reported in the articles are summarized in Table 1. The RCTs’ sample size ranged from 202 to 12,018 in the seven studies. Three studies were conducted in pediatric populations and four in adults. In three studies, TIV was the sole vaccine administered. In the remaining studies, TIV and LAIV were both used. QIV was used alone in one study and in combination with LAIV in another. Influenza virus detection was performed by PCR testing in all of the studies.

**Figure 2:**
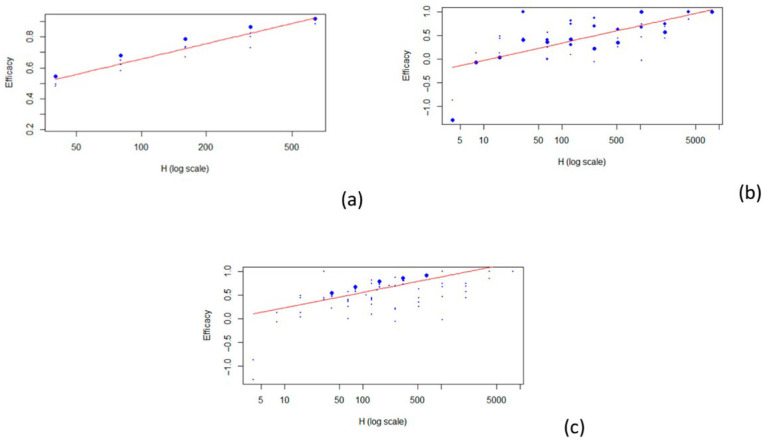
Scatterplots of vaccine efficacy and HAI titers: (**a**) children; (**b**) adults; (**c**) all. The red line represents the fitted model.

The best model that fit all the data was a logarithmic model (r^2^ = 0.397). For the 1:40 titer level, the estimate of the VE was 0.430. For the two subpopulations (children and adults), the model that best fit the data was also a logarithmic model (see Figure 2). Three articles concern the relationship between HAI titers and vaccine efficacy in children [24,25,29] and four in adults [26,27,28,30]. For the 1:40 titer level, the estimate of the VE is 0.527 for children (r^2^ = 0.859) and 0.196 for adults (r^2^ = 0.474). The results described are summarized in Table 2. Estimates for other HAI titer levels are also shown in Table 2.

### 3.1. Subgroup Analysis

To explore the sources of variation in vaccine efficacy, a subgroup analysis was performed according to the vaccine strains and type.

#### 3.1.1. Efficacy per Vaccine Strain

**Figure 3:**
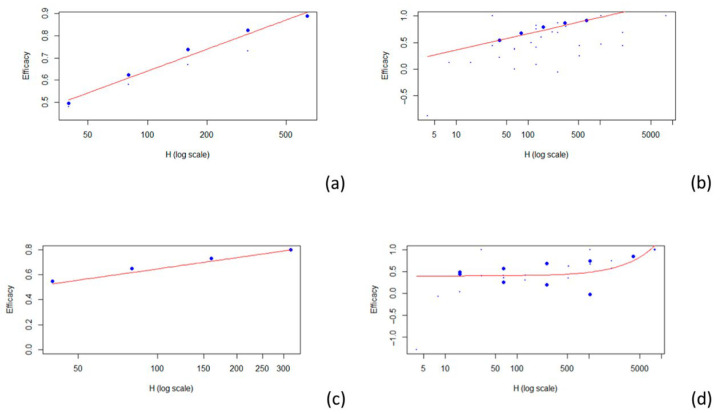
Scatterplots of vaccine efficacy and HAI titers: (**a**) A (H1N1); (**b**) A (H3N2); (**c**) B; (**d**) unspecified strain. The red line represents the fitted model.

The model that best fit the data related to the A H1N1, A H3N2, and B strains was a logarithmic model. A linear model was used for the data of unspecified strains (see Figure 3).

Four articles examine the relationship between HAI titers and efficacy against influenza A H1N1 [25,26,27,29], while four others address the same relationship for influenza A H3N2 [24,25,27,28]. Additionally, two articles focus on efficacy against influenza B [27,29], and two others discuss efficacy without specifying the involved strains [26,30]. For the 1:40 titer level, the estimated VE was 0.511 for A H1N1 (r^2^ = 0.966), 0.544 for A H3N2 (r^2^ = 0.578), 0.528 for B (r^2^ = 0.513), and 0.403 when the strains are not specified (r^2^ = 0.258). The results described are summarized in Table 3. Estimates for other HAI titer levels are presented in Table 3.

#### 3.1.2. Efficacy per Vaccine Type

**Figure 4:**
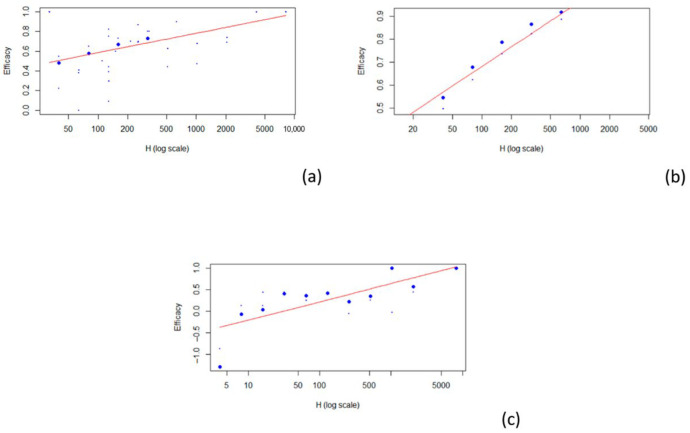
Scatterplot of vaccine efficacy and HAI titers: (**a**) TIV; (**b**) QIV; (**c**) LAIV. The red line represents the fitted model.

The best model that fit all the data involving the different type of vaccines was a logarithmic model (see Figure 4).

Five articles concern the relationship between TIV (a) HAI titers and efficacy [24,27,28,29,30], two articles concern the relationship between QIV (b) HAI titers and efficacy [25,26], and three articles concern the relationship between LAIV (c) HAI titers and efficacy [26,28,30]. For the 1:40 titer level, the estimated VE was 0.506 for TIV (r^2^ = 0.312), 0.569 for QIV (r^2^ = 0.878), and 0.048 for LAIV (r^2^ = 0.556). The results described are summarized in Table 4, and estimates for other HAI titer levels are described in Table 4.

### 3.2. Risk-of-Bias Assessment

**Figure 5:**
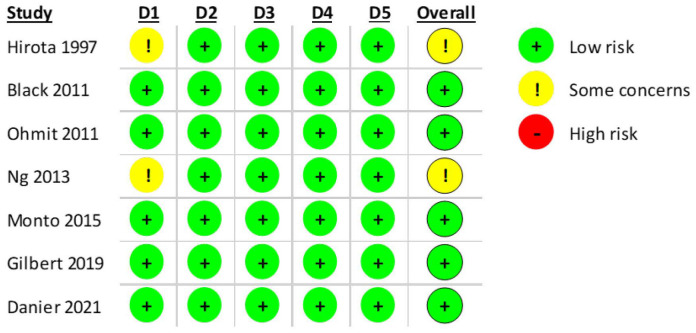
RoB2.0 assessment of the included RCT studies with 5 domains. (D1: Randomization process, D2: Deviations from the intended interventions, D3: Missing outcome data; D4: Measurement of the outcome, D5: Selection of the reported result) [24,25,26,27,28,29,30].

According to RoB2.0, five studies were classified as having a low risk of bias. However, for two of the included articles [27,29], it was not possible to verify if individuals were randomly allocated to a vaccination group or a placebo. A summary of the results is presented in Figure 5.

## 4. Discussion

This study aimed to define a model that relates the immunogenicity of the influenza vaccine to its efficacy, based on the levels of HAI titers, updating the data by Hobson et al. [13].

After extracting the data from the different articles selected, models were applied to relate the levels of HAI titers obtained to the efficacy of the vaccine and/or its protective factor against the disease. This was accomplished for the different strains as well as for the different types of vaccine. In addition, the relationship between HAI titer levels and efficacy in children was evaluated, since it has been suggested that higher HAI titer levels are necessary for a satisfactory protective factor, though there is not a consensus on this [31,32,33,34]. The adjusted model for children showed an excellent adjustment, which emphasizes the importance of HAI titers in the efficacy of the flu vaccine. The estimate for the 40 titer level is in line with the expected values. The three studies on adults coincided with the studies that included LAIVs. The efficacy values found for this vaccine were very low. The value found in adults was less than half that found in children. If we only analyzed the studies in adults that did not include LAIVs (three studies with TIVs and one study with QIV), we would obtain values of 0.587, which shows that, in this case, LAIVs negatively influence the estimates obtained for adults. As far as LAIVs are concerned, even a titer level of 400 would not meet the reference value of 0.50 (0.474). Assessing the efficacy of live attenuated influenza vaccines (LAIVs) in adults remains challenging, primarily due to their limited administration in this age group—a limitation previously underscored in a meta-analysis by Perego et al. [35]. Given the very small number of adults receiving LAIVs, even a single mismatch between the vaccine strain and the predominant circulating strain in a particular season can markedly influence efficacy estimates. In the present study, the exclusion of LAIV recipients yielded efficacy estimates consistent with those reported in the existing literature. Nonetheless, LAIVs may exhibit poor efficacy even in seasons with a good antigenic match. Mathematical modelling studies suggest that pre-existing immunity can attenuate vaccine effectiveness, potentially because memory immune responses neutralize the attenuated virus before it can trigger a robust secondary immune response [36]. This phenomenon may contribute to the higher efficacy observed in children, who are generally immunologically naive to influenza viruses. Similar findings regarding the dampening effect of pre-existing immunity on LAIV efficacy have been reported by Subbarao and colleagues [37].

A pooled analysis of influenza vaccine effectiveness stratified by population characteristics and circulating strains could not be performed due to the insufficient number of eligible studies.

Using the developed mathematical models, efficacy estimates were made for HAI titers of 30, 40, and 50. Overall, these estimates support Hobson’s data [13]. A slight increase or decrease in titer levels does not appear to significantly affect the vaccine’s efficacy.

In a recent study, the proportions of vaccine efficacy mediated by post-vaccination HAI titers were estimated to be 22% for influenza A(H1N1), 20% for influenza A(H3N2), and 37% for influenza B/Victoria [38]. The values obtained in our study are higher and are based on models that demonstrate a good adjustment. This underscores the importance of HAI titer levels in estimating the effectiveness of the flu vaccine, aligning with findings in other publications [31,32,39,40,41].

Given the suboptimal and variable efficacy of current seasonal influenza vaccines, particularly among the elderly and immunocompromised populations, the scientific community is increasingly focused on optimizing immunization strategies [18,42]. Hemagglutinin (HA) remains the primary antigenic target due to its role in viral entry and immunogenic potential. To enhance vaccine-induced protection, alternative approaches such as adjuvanted formulations, recombinant platforms, and high-dose inactivated vaccines have been introduced [43]. These strategies have demonstrated improved immunogenicity and clinical effectiveness in various populations [44,45,46].

Influenza virus infection induces both humoral and cellular immune responses, with the humoral responses usually considered as indicators of protection [40]. Antibodies targeting the HA and NA proteins, which neutralize the virus, inhibit hemagglutination, or block neuraminidase activity, are associated with protection against the disease [41]. Neuraminidase is also present in most influenza vaccines. As measured by neuraminidase inhibition (NAI) assays, anti-NA antibodies play a role independent from HAI in protection from influenza disease and/or in reducing influenza disease severity [26]. The neuraminidase inhibition component cannot be ruled out in the efficacy of the flu vaccine [47,48].

Another promising target is the matrix protein 2 ectodomain (M2e), a highly conserved region of the M2 ion channel involved in viral assembly and disassembly [49]. Despite its limited natural immunogenicity, M2e can elicit broad, IgG-mediated cross-protection when delivered in conjugated forms or as virus-like particles. However, its protective efficacy is largely dependent on Fc-mediated effector functions, and no definitive immune correlate has been established, complicating efficacy assessments outside large-scale clinical trials [50,51]. Although passive immunization with M2e-specific monoclonal antibodies has shown some efficacy in human challenge models, high-dose formulations have been associated with unacceptable levels of systemic reactogenicity [52]. As such, M2e-based vaccines are currently considered as adjuncts to HA-based formulations, particularly in cases of antigenic mismatch with circulating strains.

In addition, the nucleoprotein (NP) and other internal antigens have been explored as vaccine components capable of eliciting robust cell-mediated immune responses, including CD4^+^ and CD8^+^ T cells. These responses, particularly when involving tissue-resident memory T cells or T follicular helper cells, may significantly improve the breadth and durability of protection by targeting conserved epitopes across influenza subtypes [53,54]. Targeting a CD8^+^ T cell response in conjunction with B cell and antibody responses is particularly advantageous, and several vaccine technologies under investigation are capable of inducing such responses [55]. Despite these challenges [51,56], human studies have consistently associated elevated levels of cross-reactive CD8^+^ T cells with reduced viral shedding and milder clinical disease, reinforcing their importance as a target in next-generation vaccine development [55,57,58].

Despite the valuable insights provided by our modelling approach, the study has several limitations that warrant consideration. The first is that only seven studies fulfilled the inclusion criteria, which limited the scope of the analysis, particularly in subgroup comparisons and strain-specific assessments. The limited number of eligible studies also prevented pooled analyses stratified by host characteristics or circulating viral strains. The evaluation of live attenuated influenza vaccines (LAIVs) in adults posed additional challenges, as their use in this age group is uncommon, and the small sample size makes the efficacy estimates highly sensitive to factors such as strain mismatch in a given season. Moreover, while the model focused on HAI titers as a correlate of protection, it did not account for other immune markers as components of influenza vaccine-induced immunity. Finally, the use of mathematical modelling inherently involves assumptions and simplifications that may not fully capture the biological complexity of immune responses, particularly in populations with varied levels of prior exposure to influenza viruses. These limitations should be considered when interpreting our findings.

## 5. Conclusions

In conclusion, this study reinforces the role of HAI titers as a key correlate of protection in influenza vaccination, particularly in children, in whom higher antibody levels appear necessary to achieve adequate efficacy. The modelling results demonstrated a clear association between increasing HAI titers and improved vaccine performance, especially for inactivated formulations such as TIV and QIV. Conversely, the substantially lower efficacy observed for LAIVs in adults, likely influenced by limited data availability and underlying factors such as pre-existing immunity, highlights the need for further targeted research in this subgroup. While the models presented offer valuable insights, they are based on a restricted pool of studies and focused primarily on humoral markers. Future vaccine developers and evaluators should consider these findings, while also integrating additional immunological and population-specific factors.

## Figures and Tables

**Table 1:** Summary of the included RCT studies. Abbreviations: TIV, trivalent inactivated vaccine; QIV, tetravalent inactivated vaccine; LAIV, live attenuated vaccine; PCR, polymerase chain reaction; y, years; m, months; N, sample size.

Author	Country	N	Population	Season	Vaccine	Test
Hirota 1997 [27]	Japan	202	Adults (19–76 y)	1991/1992	TIV	PCR
Black 2011 [24]	Europe and USA	777	Children (6–17 m)	2007 to 2009	TIV	PCR
Ohmit 2011 [30]	USA	5510	Adults (18–49 y)	2007/2008	TIV and LAIV	PCR
Ng 2013 [29]	Hong Kong (China)	3092	Children (6–17 m)	2010	TIV	PCR
Monto 2015 [28]	USA	497	Adults (18–49 y)	2007/2008	TIV and LAIV	PCR
Gilbert 2019 [26]	USA	1952	Adults (18–49 y)	2007/2008	QIV and LAIV	PCR
Danier 2021 [25]	USA and UK	12,018	Children (6–35 m)	2011 to 2014	QIV	PCR

**Table 2:** Comparison of vaccine efficacy in different populations according to several HAI levels. Abbreviations: r^2^, coefficient of determination; VE, vaccine efficacy; HAI, hemagglutination inhibition; CI, confidence interval.

Population	Model	r^2^	VE for HAI Level 30 (95% CI)	VE for HAI Level 40 (95% CI)	VE for HAI Level 50 (95% CI)	Proportion of Studies with Low Risk of Bias
Children	Logarithmic	0.859	0.486 (0.438–0.534)	0.527 (0.486–0569)	0.559 (0.522–0.596)	2/3
Adults	Logarithmic	0.474	0.150 (0.020–0.279)	0.196 (0.075–0.317)	0.231 (0.117–0.346)	3/4
All	Logarithmic	0.397	0.389 (0.301–0.476)	0.430 (0.351–0.509)	0.461 (0.388–0.535)	5/7

**Table 3:** Comparison of vaccine efficacy by vaccine strain according to several HAI levels.

Criteria	Model	r^2^	VE for HAI Level 30 (95% CI)	VE for HAI Level 40 (95% CI)	VE for HAI Level 50 (95% CI)	Proportion of Studies with Low Risk of Bias
Influenza A (H1N1)	Logarithmic	0.966	0.470 (0.440–0.501)	0.511 (0.485–0.538)	0.543 (0.519–0.567)	2/4
Influenza A (H3N2)	Logarithmic	0.5778	0.506 (0.426–0.586)	0.544 (0.474–0.615)	0.574 (0.511–0.637)	3/4
Influenza B	Logarithmic	0.5133	0.490 (0.221–0.759)	0.528 (0.301–0.754)	0.557 (0.361–0.753)	0/2
Unspecified strain	Linear	0.2579	0.402 (0.286–0.519)	0.403 (0.287–0.519)	0.404 (0.288–0.520)	2/2

Abbreviations: r^2^, coefficient of determination; VE, vaccine efficacy; HAI, hemagglutination inhibition; CI, confidence interval.

**Table 4:** Comparison of vaccine efficacy by vaccine types according to several HAI levels.

Criteria	Model	r^2^	VE for HAI Level 30 (95% CI)	VE for HAI Level 40 (95% CI)	VE for HAI Level 50 (95% CI)	Proportion of Studies with Low Risk of Bias
TIV	Logarithmic	0.312	0.481 (0.381–0.582)	0.506 (0.415–0.597)	0.525 (0.441–0.609)	3/5
QIV	Logarithmic	0.8784	0.533 (0.478–0.588)	0.569 (0.520–0.617)	0.596 (0.553–0.639)	2/2
LAIV	Logarithmic	0.5563	−0.006 (−0.189–0.178)	0.0476 (−0.126–0.221)	0.089 (−0.078–0.255)	3/3

Abbreviations: r^2^, coefficient of determination; HAI, hemagglutination inhibition; VE, vaccine efficacy; CI, confidence interval; TIV, trivalent inactivated vaccine; QIV, tetravalent inactivated vaccine; LAIV, live attenuated vaccine.

## Data Availability

The original contributions presented in this study are included in the article. Further inquiries can be directed to the corresponding authors.

## References

[1] European Centre for Disease Prevention and Control (2020). Systematic Review of the Efficacy, Effectiveness and Safety of Newer and Enhanced Seasonal Influenza Vaccines for the Prevention of Laboratory-Confirmed Influenza in Individuals Aged 18 Years and Over.

[2] WHO The Global Influenza Surveillance and Response System. https://www.who.int/initiatives/global-influenza-surveillance-and-response-system.

[3] (2020). WHO. WHO Regional Office for Europe Recommendations on Influenza Vaccination for the 2020/2021 Season During the Ongoing COVID-19 Pandemic.

[4] Buchy P., Badur S. (2020). Who and When to Vaccinate Against Influenza. Int. J. Infect. Dis..

[5] Luo M., Rossmann M.G., Rao V.B. (2012). Influenza Virus Entry. Viral Molecular Machines.

[6] Hutchinson E.C. (2018). Influenza Virus. Trends Microbiol..

[7] Bouvier N.M., Palese P. (2008). The Biology of Influenza Viruses. Vaccine.

[8] Kumar V. (2017). Influenza in Children. Indian J. Pediatr..

[9] Keshavarz M., Mirzaei H., Salemi M., Momeni F., Mousavi M.J., Sadeghalvad M., Arjeini Y., Solaymani-Mohammadi F., Sadri Nahand J., Namdari H. (2019). Influenza Vaccine: Where Are We and Where Do We Go?. Rev. Med. Virol..

[10] Kumar A., Meldgaard T.S., Bertholet S. (2018). Novel Platforms for the Development of a Universal Influenza Vaccine. Front. Immunol..

[11] Krammer F. (2015). Emerging Influenza Viruses and the Prospect of a Universal Influenza Virus Vaccine. Biotechnol. J..

[12] Sridhar S., Brokstad K., Cox R. (2015). Influenza Vaccination Strategies: Comparing Inactivated and Live Attenuated Influenza Vaccines. Vaccines.

[13] Hobson D., Curry R.L., Beare A.S., Ward-Gardner A. (1972). The Role of Serum Haemagglutination-Inhibiting Antibody in Protection Against Challenge Infection with Influenza A2 and B Viruses. Epidemiol. Infect..

[14] Stephenson I., Heath A., Major D., Newman R.W., Hoschler K., Junzi W., Katz J.M., Weir J.P., Zambon M.C., Wood J.M. (2009). Reproducibility of Serologic Assays for Influenza Virus A (H5N1). Emerg. Infect. Dis..

[15] Wagner R., Göpfert C., Hammann J., Neumann B., Wood J., Newman R., Wallis C., Alex N., Pfleiderer M. (2012). Enhancing the Reproducibility of Serological Methods Used to Evaluate Immunogenicity of Pandemic H1N1 Influenza Vaccines—An Effective EU Regulatory Approach. Vaccine.

[16] Wood J.M., Major D., Heath A., Newman R.W., Höschler K., Stephenson I., Clark T., Katz J.M., Zambon M.C. (2012). Reproducibility of Serology Assays for Pandemic Influenza H1N1: Collaborative Study to Evaluate a Candidate WHO International Standard. Vaccine.

[17] Martins J.P., Santos M., Martins A., Felgueiras M., Santos R. (2023). Seasonal Influenza Vaccine Effectiveness in Persons Aged 15–64 Years: A Systematic Review and Meta-Analysis. Vaccines.

[18] Belongia E.A., Simpson M.D., King J.P., Sundaram M.E., Kelley N.S., Osterholm M.T., McLean H.Q. (2016). Variable Influenza Vaccine Effectiveness by Subtype: A Systematic Review and Meta-Analysis of Test-Negative Design Studies. Lancet Infect. Dis..

[19] Rohatgi A. WebPlotDigitizer User Manual Version 3.4. https://web.eecs.utk.edu/~dcostine/personal/PowerDeviceLib/DigiTest/index.html.

[20] R Core Team The R Project for Statistical Computing. http://www.R-project.org/.

[21] Wang W., Yan J. (2021). Shape-Restricted Regression Splines with R Package Splines2. J. Data Sci..

[22] Wood S., Pya N., Säfken B. (2016). Smoothing Parameter and Model Selection for General Smooth Models (with Discussion). J. Am. Stat. Assoc..

[23] Sterne J.A., Hernán M.A., Reeves B.C., Savović J., Berkman N.D., Viswanathan M., Henry D., Altman D.G., Ansari M.T., Boutron I. (2016). ROBINS-I: A Tool for Assessing Risk of Bias in Non-Randomised Studies of Interventions. BMJ.

[24] Black S., Nicolay U., Vesikari T., Knuf M., Del Giudice G., Della Cioppa G., Tsai T., Clemens R., Rappuoli R. (2011). Hemagglutination Inhibition Antibody Titers as a Correlate of Protection for Inactivated Influenza Vaccines in Children. Pediatr. Infect. Dis. J..

[25] Danier J., Callegaro A., Soni J., Carmona A., Kosalaraska P., Rivera L., Friel D., Pu W., Vantomme V., Dbaibo G. (2022). Association Between Hemagglutination Inhibition Antibody Titers and Protection Against Reverse-Transcription Polymerase Chain Reaction–Confirmed Influenza Illness in Children 6–35 Months of Age: Statistical Evaluation of a Correlate of Protection. Open Forum Infect. Dis..

[26] Gilbert P.B., Fong Y., Juraska M., Carpp L.N., Monto A.S., Martin E.T., Petrie J.G. (2019). HAI and NAI Titer Correlates of Inactivated and Live Attenuated Influenza Vaccine Efficacy. BMC Infect. Dis..

[27] Hirota Y. (1996). The Hemagglutination Inhibition Antibody Responses to an Inactivated Influenza Vaccine Among Healthy Adults: With Special Reference to the Prevaccination Antibody and Its Interaction with Age. Vaccine.

[28] Monto A.S., Petrie J.G., Cross R.T., Johnson E., Liu M., Zhong W., Levine M., Katz J.M., Ohmit S.E. (2015). Antibody to Influenza Virus Neuraminidase: An Independent Correlate of Protection. J. Infect. Dis..

[29] Ng S., Fang V.J., Ip D.K.M., Chan K.-H., Leung G.M., Peiris J.S.M., Cowling B.J. (2013). Estimation of the Association Between Antibody Titers and Protection Against Confirmed Influenza Virus Infection in Children. J. Infect. Dis..

[30] Ohmit S.E., Petrie J.G., Cross R.T., Johnson E., Monto A.S. (2011). Influenza Hemagglutination-Inhibition Antibody Titer as a Correlate of Vaccine-Induced Protection. J. Infect. Dis..

[31] Tsang T.K., Cauchemez S., Perera R.A.P.M., Freeman G., Fang V.J., Ip D.K.M., Leung G.M., Malik Peiris J.S., Cowling B.J. (2014). Association Between Antibody Titers and Protection Against Influenza Virus Infection Within Households. J. Infect. Dis..

[32] Coudeville L., Bailleux F., Riche B., Megas F., Andre P., Ecochard R. (2010). Relationship Between Haemagglutination-Inhibiting Antibody Titres and Clinical Protection Against Influenza: Development and Application of a Bayesian Random-Effects Model. BMC Med. Res. Methodol..

[33] Cox R. (2013). Correlates of Protection to Influenza Virus, Where Do We Go from Here?. Hum. Vaccines Immunother..

[34] Belshe R.B., Gruber W.C., Mendelman P.M., Mehta H.B., Mahmood K., Reisinger K., Treanor J., Zangwill K., Hayden F.G., Bernstein D.I. (2000). Correlates of Immune Protection Induced by Live, Attenuated, Cold-Adapted, Trivalent, Intranasal Influenza Virus Vaccine. J. Infect. Dis..

[35] Perego G., Vigezzi G.P., Cocciolo G., Chiappa F., Salvati S., Balzarini F., Odone A., Signorelli C., Gianfredi V. (2021). Safety and Efficacy of Spray Intranasal Live Attenuated Influenza Vaccine: Systematic Review and Meta-Analysis. Vaccines.

[36] Matrajt L., Halloran M.E., Antia R. (2020). Successes and Failures of the Live-Attenuated Influenza Vaccine: Can We Do Better?. Clin. Infect. Dis..

[37] Subbarao K. (2021). Live Attenuated Cold-Adapted Influenza Vaccines. Cold Spring Harb. Perspect. Med..

[38] Lim W.W., Feng S., Wong S.-S., Sullivan S.G., Cowling B.J. (2024). Hemagglutination Inhibition Antibody Titers as Mediators of Influenza Vaccine Efficacy Against Symptomatic Influenza A(H1N1), A(H3N2), and B/Victoria Virus Infections. J. Infect. Dis..

[39] Skowronski D.M., Moser F.S., Janjua N.Z., Davoudi B., English K.M., Purych D., Petric M., Pourbohloul B. (2013). H3N2v and Other Influenza Epidemic Risk Based on Age-Specific Estimates of Sero-Protection and Contact Network Interactions. PLoS ONE.

[40] Beran J., Wertzova V., Honegr K., Kaliskova E., Havlickova M., Havlik J., Jirincova H., Van Belle P., Jain V., Innis B. (2009). Challenge of Conducting a Placebo-Controlled Randomized Efficacy Study for Influenza Vaccine in a Season with Low Attack Rate and a Mismatched Vaccine B Strain: A Concrete Example. BMC Infect. Dis..

[41] Cowling B.J., Lim W.W., Perera R.A.P.M., Fang V.J., Leung G.M., Peiris J.S.M., Tchetgen Tchetgen E.J. (2019). Influenza Hemagglutination-Inhibition Antibody Titer as a Mediator of Vaccine-Induced Protection for Influenza B. Clin. Infect. Dis..

[42] Osterholm M.T., Kelley N.S., Sommer A., Belongia E.A. (2012). Efficacy and Effectiveness of Influenza Vaccines: A Systematic Review and Meta-Analysis. Lancet Infect. Dis..

[43] Taaffe J., Ostrowsky J.T., Mott J., Goldin S., Friede M., Gsell P., Chadwick C. (2024). Advancing Influenza Vaccines: A Review of next-Generation Candidates and Their Potential for Global Health Impact. Vaccine.

[44] DiazGranados C.A., Dunning A.J., Kimmel M., Kirby D., Treanor J., Collins A., Pollak R., Christoff J., Earl J., Landolfi V. (2014). Efficacy of High-Dose Versus Standard-Dose Influenza Vaccine in Older Adults. N. Engl. J. Med..

[45] Frey S.E., Reyes M.R.A.-D.L., Reynales H., Bermal N.N., Nicolay U., Narasimhan V., Forleo-Neto E., Arora A.K. (2014). Comparison of the Safety and Immunogenicity of an MF59^®^-Adjuvanted with a Non-Adjuvanted Seasonal Influenza Vaccine in Elderly Subjects. Vaccine.

[46] Domnich A., Arata L., Amicizia D., Puig-Barberà J., Gasparini R., Panatto D. (2017). Effectiveness of MF59-Adjuvanted Seasonal Influenza Vaccine in the Elderly: A Systematic Review and Meta-Analysis. Vaccine.

[47] Memoli M.J., Shaw P.A., Han A., Czajkowski L., Reed S., Athota R., Bristol T., Fargis S., Risos K., Powers J.H. (2016). Evaluation of Antihemagglutinin and Antineuraminidase Antibodies as Correlates of Protection in an Influenza A/H1N1 Virus Healthy Human Challenge Model. mBio.

[48] Eichelberger M.C., Monto A.S. (2019). Neuraminidase, the Forgotten Surface Antigen, Emerges as an Influenza Vaccine Target for Broadened Protection. J. Infect. Dis..

[49] Wei C.-J., Crank M.C., Shiver J., Graham B.S., Mascola J.R., Nabel G.J. (2020). Next-Generation Influenza Vaccines: Opportunities and Challenges. Nat. Rev. Drug Discov..

[50] Mozdzanowska K., Feng J., Eid M., Kragol G., Cudic M., Otvos L., Gerhard W. (2003). Induction of Influenza Type A Virus-Specific Resistance by Immunization of Mice with a Synthetic Multiple Antigenic Peptide Vaccine That Contains Ectodomains of Matrix Protein 2. Vaccine.

[51] Wang R., Song A., Levin J., Dennis D., Zhang N., Yoshida H., Koriazova L., Madura L., Shapiro L., Matsumoto A. (2008). Therapeutic Potential of a Fully Human Monoclonal Antibody Against Influenza A Virus M2 Protein. Antivir. Res..

[52] Staneková Z., Varečková E. (2010). Conserved Epitopes of Influenza A Virus Inducing Protective Immunity and Their Prospects for Universal Vaccine Development. Virol. J..

[53] Sridhar S., Begom S., Bermingham A., Hoschler K., Adamson W., Carman W., Bean T., Barclay W., Deeks J.J., Lalvani A. (2013). Cellular Immune Correlates of Protection Against Symptomatic Pandemic Influenza. Nat. Med..

[54] Wilkinson T.M., Li C.K.F., Chui C.S.C., Huang A.K.Y., Perkins M., Liebner J.C., Lambkin-Williams R., Gilbert A., Oxford J., Nicholas B. (2012). Preexisting Influenza-Specific CD4+ T Cells Correlate with Disease Protection Against Influenza Challenge in Humans. Nat. Med..

[55] Leong S.L., Gras S., Grant E.J. (2024). Fighting Flu: Novel CD8^+^ T-Cell Targets Are Required for Future Influenza Vaccines. Clin. Trans. Immunol..

[56] Yewdell J.W., Bennink J.R. (1999). Immunodominance in major histocompatibility complex class I–restricted T lymphocyte responses. Annu. Rev. Immunol..

[57] Lee L.Y.-H., Ha D.L.A., Simmons C., De Jong M.D., Chau N.V.V., Schumacher R., Peng Y.C., McMichael A.J., Farrar J.J., Smith G.L. (2008). Memory T Cells Established by Seasonal Human Influenza A Infection Cross-React with Avian Influenza A (H5N1) in Healthy Individuals. J. Clin. Investig..

[58] Kreijtz J.H.C.M., Fouchier R.A.M., Rimmelzwaan G.F. (2011). Immune Responses to Influenza Virus Infection. Virus Res..

